# Long-term development of loneliness in older people and associations with stringency of COVID-19 measures: an observational cohort study

**DOI:** 10.1093/ageing/afae069

**Published:** 2024-04-10

**Authors:** Flurina Meier Schwarzer, Nicole Probst-Hensch, Marek Kwiatkowski, Marc Höglinger

**Affiliations:** Winterthur Institute of Health Economics, School of Management and Law, Zurich University of Applied Sciences, Winterthur 8401, Switzerland; Department of Epidemiology and Public Health, Swiss Tropical and Public Health Institute, Allschwil 4123, Switzerland; University of Basel, Basel 4003, Switzerland; Department of Epidemiology and Public Health, Swiss Tropical and Public Health Institute, Allschwil 4123, Switzerland; University of Basel, Basel 4003, Switzerland; Department of Epidemiology and Public Health, Swiss Tropical and Public Health Institute, Allschwil 4123, Switzerland; University of Basel, Basel 4003, Switzerland; Winterthur Institute of Health Economics, School of Management and Law, Zurich University of Applied Sciences, Winterthur 8401, Switzerland

**Keywords:** social isolation, pandemic, vulnerable subgroups, longitudinal study, older people

## Abstract

**Background:**

Most longitudinal studies found heightened feelings of loneliness in older people in spring 2020 compared to times before the pandemic. However, longer-term effects are more disputed. We, therefore, investigated changes in loneliness in older people throughout the first 21 months of the COVID-19 pandemic in Switzerland and examined the association between the stringency of COVID-19 measures and feelings of loneliness.

**Methods:**

We assessed loneliness (3-item University of California Los Angeles (UCLA) loneliness scale) in a nationally representative longitudinal observational online survey. Older people (65–79 years) were surveyed between March 2020 and December 2021. Proportions of people feeling lonely (UCLA loneliness scale >6) were compared in two stringent phases (‘lockdown’, Oxford University stringency index ≥55) and two less restricted phases. Additionally, we explored the situation of potentially more susceptible subgroups (living alone, women, chronic and mental diseases, low educational level and low income).

**Results:**

Phases with stringent measures were associated with higher levels of loneliness in older people. People living alone, women, people with noncommunicable or mental disease diagnoses and lower income show consistently higher levels of loneliness. However, the differences are not accentuated in phases with more stringent measures. We found little differences between subgroups with varying educational levels.

**Conclusions:**

Even in a country with relatively less stringent COVID-19 measures like Switzerland, an increase in the proportion of older people that feel lonely could be found during phases with more stringent COVID-19 measures. Lockdown phases should, therefore, be accompanied by evidence-based interventions to relieve loneliness to avoid adverse short- and long-term consequences.

## Key Points

- Phases with more stringent COVID-19 measures (lockdowns) were associated with higher levels of loneliness in older people.
- Even lenient COVID-19 measures were associated with a substantial increase in numbers of older people that felt lonely.
- Women, people living alone, with noncommunicable diseases (NCD) or mental disease and lower income show consistently higher levels of loneliness.
- Especially the early phases of lockdowns should be accompanied by interventions to relieve loneliness.

## Introduction

Loneliness has been associated with adverse health outcomes in older people such as mortality, coronary heart disease, stroke, depression and dementia and is a predictor for nursing home entries [1–5]. Therefore, loneliness has become an increasing concern in the care for community-dwelling older people. Since spring 2020, these concerns have been exacerbated due to the coronavirus disease (COVID-19) pandemic and the governmental measures to counter its spread which older people were especially affected by because they were particularly at risk of serious complications after an infection.

Several studies investigated whether loneliness in older people increased during COVID-19 lockdowns. Most longitudinal studies that compared the lockdown in spring 2020 to the time before the COVID-19 pandemic found that loneliness increased in the older people [6–12]. Only few longitudinal studies found no such change in loneliness or only in subgroups [13, 14].

However, longer-term effects on loneliness throughout the pandemic are more disputed, and it is unclear whether there has been an attenuation of loneliness as people may have developed strategies to deal with the imposed restrictions and prevent feelings of loneliness in the longer term. Some studies report an attenuation of feelings of loneliness throughout the first weeks or months of the pandemic [9, 10, 15, 16], whereas another study reports an increase throughout the first month of the pandemic [12]. These changes might be influenced by the stringency of COVID-19 measures that varied throughout the first month of the pandemic. COVID-19 measures as well as the extent to which physical or social isolation mandates were adhered to have been found to be associated with more prevalent feelings of loneliness [10, 11, 14, 17, 18]. However, to our knowledge no study examined the relationship between loneliness in older people and changes in the stringency of COVID-19 measures in a continuous manner over several months.

According to the Oxford University stringency index, Switzerland implemented relatively liberal measures to reduce the spread of COVID-19 compared to other Western European countries [19]. The mean score of the Oxford stringency index for the period March 2020 to December 2021 was 51 out of 100 possible points for Switzerland [19]. Most other Western European countries like Austria (60), Belgium (55), France (58), Germany (61), Greece (69), Ireland (63), Italy (69), the Netherlands (59), Portugal (62), Spain (60) and the United Kingdom (62) had a higher mean score throughout this period. Switzerland’s score was similar to Northern European countries such as Denmark (50), Norway (50) or Sweden (52) but higher than Finland (44) and Luxembourg (48). Also, when looking not at the mean but at peak levels Switzerland’s score was lower than the mentioned countries except for Denmark, Finland and Sweden during the first wave and Finland during the second wave.

In our study, we investigated the long-term development of feelings of loneliness and its associations with the stringency of COVID-19 measures in older people over a period of 21 months, i.e. between March 2020 and December 2021 in Switzerland, a country with relatively less stringent COVID-19 measures. Furthermore, we scrutinised several subgroups that have been found to be particularly susceptible to loneliness.

## Methods

### Data source and study sample

We used data from the Swiss COVID-19 Social Monitor survey, a nationally representative cohort study established in March 2020 and ongoing until November 2022 [20]. Participants were randomly selected from an existing online panel pool that had been actively recruited by a Survey Company (LINK Institute, Zurich, Switzerland) using random sampling based on landline phone directories and randomly generated mobile numbers. The sample was stratified by age, sex, and language regions.

For the present analyses, we restricted the sample to participants aged 65 to 79 years. We used data from the first 20 waves of the survey, covering the timespan of March 2020 (first lockdown) to December 2021. During this time period, participants were surveyed online every 2 to 5 weeks. The resulting sample comprised 303 participants from wave 1 on, and another 177 from wave 12 (December 2020) on, when a refreshment sample was added.

### Outcome

We measured loneliness with the 3-item University of California Los Angeles (UCLA) loneliness scale [21], covering feelings of lacking companionship, of being isolated and of being left out. Hughes *et al.* (2004) [21] found a Cronbach’s alpha of 0.72 for the 3-item UCLA loneliness scale in their validation study. Our study’s mean Cronbach’s alpha over all waves was 0.81, which implies good internal consistency. We used a cut-off point of 6 for the resulting 3 to 9 score to categorise respondents into ‘feeling lonely’ as it was the cut-off point that was most often used in the literature [22, 23]. Because this instrument was only added to the survey in the second wave (6 April 2020), data is shown from this date onwards.

### Explanatory variable

We used the Oxford University stringency index to operationalise the stringency of COVD-19 restriction measures [24]. We a priori divided the observational period into phases with more stringent measures (stringency index ≥55) and less stringent measures (stringency index <55). This cut-off point could not be based on any existing predefined standards. It was set higher than the median of 27 European countries between June and August 2020, which was at 46.1 and was used by Wester *et al.* (2022). The application of the cut-off point resulted in four phases: two with more stringent measures (phase I, ‘first lockdown’: 30.03.20–05.06.2020; waves 1–6 and phase III, ‘second lockdown’: 02.11.2020–18.04.2021, waves 11–15) and two with less stringent measures (phase II, ‘first open phase’: 06.06.20–01.11.2020; waves 7–10 and phase IV, ‘second open phase’: 19 April 2021–31 December 2021; waves 16–20).

### Subgroup analyses

Several subgroups have been shown in previous research to be more susceptible to loneliness during the COVID-19 pandemic, e.g. women, people living alone with noncommunicable diseases (NCD), mental diseases or lower income [7, 9–12, 14, 16]. The susceptibility in people with lower educational levels is more disputed [9, 11, 14, 25]. We, therefore, a priori selected the following subgroups to investigate the modification of the association between stringency of COVID-19 measures and loneliness: living alone (yes/no); gender (m/w); presence of NCD diagnosis at any time in their lifetime (yes/no); presence of mental disease diagnosis at any time in their lifetime (yes/no); educational level (compulsory education, secondary degree, tertiary degree); household income (Swissfrancs (CHF) <5,000/month, CHF 5,000-9,999/month, CHF ≥10,000/month).

### Statistical analyses

We present means and standard deviations or percentages to describe our study population and their background variables at baseline in wave 1 as well as after the addition of the refreshment sample in wave 12. Differences between the two were assessed using chi-squared tests for categorical and paired t-tests for continuous variables. The development of loneliness throughout the first 21 months of the COVID-19 pandemic is represented with means of the UCLA loneliness scale (range: 3 to 9) and 95% confidence intervals (CI) for each wave.

To analyse the differences in loneliness between phases with more stringent and phases with less stringent measures, we described the differences in proportions and 95% CI between the four phases with different stringency measures. Proportions are from the pooled observations of a phase. Standard errors clustered on participant identification were used throughout. We used weights to make the sample representative of the Swiss census population in 2018 with regard to age, gender and language region (online supplement of [26]). Due to very low missing rates in the main outcome over all waves (<5%), no imputation strategy was applied. We used Stata 17.0 for all analyses.

## Results

Mean response rates were 85% (wave 1 to wave 11) and 89% (wave 12 to wave 20). At baseline, our study population had a mean age of 71.1 years (standard deviation (SD): 4.0) and 45% of participants were women (Table 1). The addition of the refreshment sample did not result in significant or relevant changes in sample characteristics.

**Table 1:** Descriptive statistics of the study sample at baseline (wave 1) and after the addition of the refreshment sample (wave 12)

		Wave 1 (N = 303)	Wave 12 (N = 480)	Group diff.
Characteristic		N (%), mean (SD)	N (%), mean (SD)	(*P*-value)
*Age*		71.1 (4.0)	71.4 (4.2)	0.385
*Living situation*				0.371
	Living alone	77 (25%)	126 (28%)	
	Not living alone	226 (75%)	344 (72%)	
*Gender*				0.769
	Women	135 (45%)	219 (46%)	
	Men	168 (55%)	261 (54%)	
*NCD diagnosis*				0.387
	Yes	198 (66%)	329 (69%)	
	No	104 (34%)	151 (31%)	
*Psychological diagnosis*				0.915
	Yes	22 (7%)	34 (7%)	
	No	280 (92%)	446 (93%)	
	No answer	1 (0.3%)	0 (0%)	
*Educational level*				0.958
	Compulsory education	19 (6%)	30 (6%)	
	Secondary degree	230 (76%)	361 (75%)	
	Tertiary degree	53 (17%)	88 (18%)	
*Household income*				0.980
	CHF <5,000/month	97 (32%)	160 (33%)	
	CHF 5,000–9,999/month	144 (48%)	225 (47%)	
	CHF >10,000/month	38 (13%)	57 (12%)	

N: Numbers; NCD: noncommunicable diseases; SD: Standard deviation

**Figure 1:**
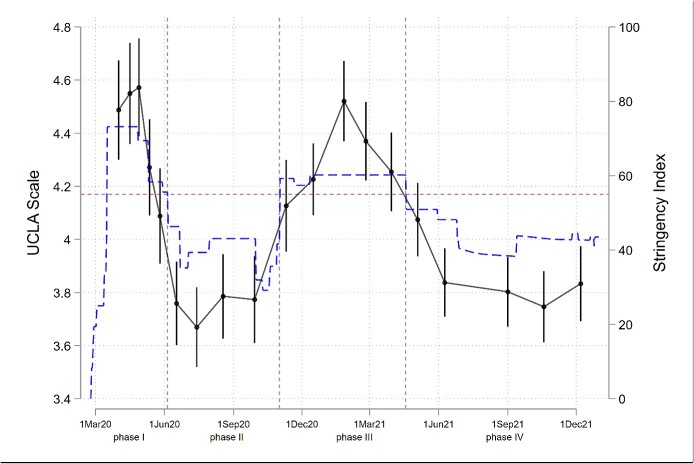
UCLA loneliness scale over the first 21 months of the pandemic and Oxford University stringency index. Phases I and III: more stringent: Oxford University stringency index: ≥55; phases II and IV: less stringent: Oxford University stringency index: <55. The blue dashed line shows the values of the Oxford University stringency index; the purple line with shorter dashes shows the Oxford University stringency index cut-off point of 55.

The UCLA loneliness scale showed substantial variation over the first 21 months of the pandemic (Figure 1). It was highest in wave 4 (start: 27.04.2020), where it reached 4.57 [95%-CI: 4.39; 4.75] and lowest in wave 8 (start: 13.07.2020) with 3.67 [3.52; 3.82]. Loneliness was generally higher during lockdown periods (phase I and III) compared to phases with less stringent measures (phases II and IV). During both lockdowns, the level of loneliness decreased after reaching a peak (difference wave 4 versus 6: -0.48, [CI: −0.23; −0.74]; wave 13 versus wave 15: −0.27 [−0.05; −0.48]. Furthermore, during the second lockdown, we observed a sharp increase in loneliness at the beginning of the lockdown (difference wave 11 versus 13: 0.39 [0.63; 0.16]. We have no information about any potential similar rise at the beginning of the first lockdown because our measurement only started when loneliness was already close to its peak in phase I.

**Figure 2:**
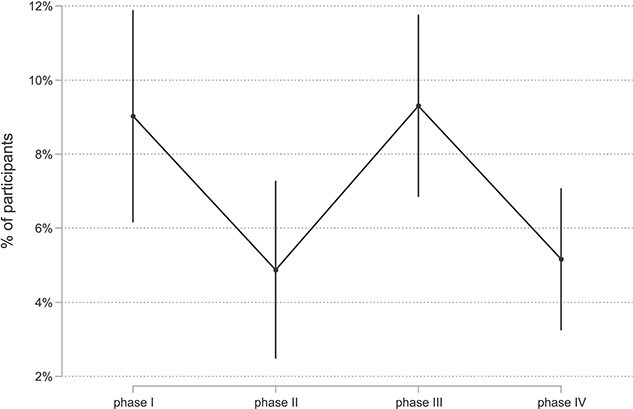
High feelings of loneliness (UCLA loneliness scale >6) in four phases with more versus less stringent measures. Phases I and III: more stringent: Oxford University stringency index: ≥55; phases II and IV: less stringent: Oxford University stringency index: <55.

Even though the measures in the second lockdown were less strict compared to the first, levels of loneliness were slightly higher in the second lockdown (mean of second lockdown: 9.3% CI: 7.1%—12.1%) than in the first (mean of first lockdown: 9.0% CI: 6.5%—12.3%). The difference between the two phases is small, however (Figure 2; difference between first and second lockdown *P* = 0.805). During both lockdown phases, the proportion of people feeling lonely was significantly higher compared to the first open phase (mean first open phase: 4.9% CI: 3.0%—7.9%; difference to both lockdown phases *P* < 0.001) as well as to the second open phase (mean second open phase: 5.2% CI: 3.6%—7.4%; difference to first lockdown *P* = 0.005, to second lockdown *P* < 0.001).

**Figure 3:**
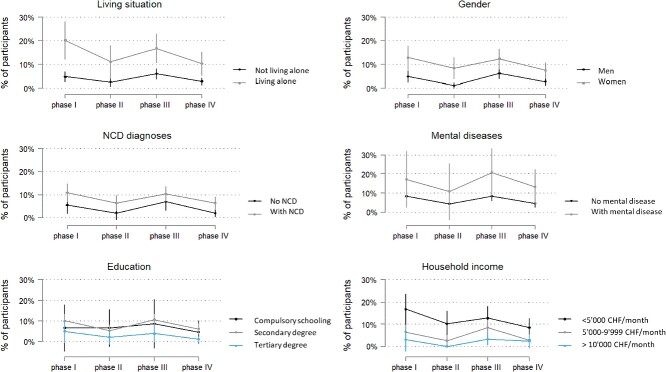
High feelings of loneliness (UCLA loneliness scale >6) according to the four phases of more versus less stringent measures by subgroups. Phases I and III: more stringent: Oxford University stringency index: ≥55; phases II and IV: less stringent: Oxford University stringency index: <55.

People living alone reported higher levels of loneliness than people living with someone throughout all four phases (Figure 3). The same could be found in other subgroups such as women, people with NCD diagnoses and people with mental disease diagnoses. Concerning education, virtually no difference in loneliness could be found between people with compulsory schooling and those with secondary degrees. People with tertiary degrees were slightly less lonely than the other two subgroups over all phases. People with low household income (CHF <5,000/month) felt loneliest throughout all stringency phases compared to people with middle (CHF 5,000–9,999/month) or high income (CHF ≥10,000/month).

With few exceptions, the development over the four stringency phases show no clear differences between subgroups, meaning that almost all subgroups show a similar pattern of change over time, with higher levels of loneliness in phases with more stringent measures and lower levels of loneliness in phases with less stringent measures (Figure 3). The relative differences between more and less susceptible groups remains constant during all phases. Exceptions are people not living alone, those in the highest education group, and those with a household income of CHF ≥10,000/month, who seemed less reactive both in absolute and in relative terms.

## Discussion

Our analyses show that throughout the first 21 months of the COVID-19 pandemic in Switzerland, a larger share of the older population felt lonely in phases with more stringent measures compared to phases with less stringent measures. These findings corroborate results from other studies which found associations between the severity of restriction measures and higher feelings of loneliness in the older population [10, 11, 17]. Compared to most other Western European countries Switzerland had less stringent measures when measured by the Oxford University stringency index during most of the COVID-19 pandemic [19]. We, therefore, expect that the found effect could be even stronger in other countries with more stringent measures.

Within both lockdown periods, levels of loneliness varied considerably. They were high at the start of our measurements or increased shortly after the beginning of the second lockdown and thendecreased after reaching a peak. Several other studies have found a decrease in loneliness within the first weeks or months of the first COVID-19 lockdown [9, 10, 15, 16]. In some cases, this decrease in loneliness was accompanied by an easing of restriction measures. In our study, however, this was only the case in the first lockdown, not in the second. This might suggest that at least within a lockdown phase, older people are somewhat able to adapt to the circumstances. As we focused on a formal measure of stringency, this adaptation might be due to differences in compliance with measures over time.

We found little difference in loneliness levels between the first and second lockdown phase, even though the second lockdown had relatively less stringent restriction measures than the first. This suggests that there was little adaptation to lockdown measures that could ease the effect of a second lockdown on feelings of loneliness. However, other factors could have been influenced by any of these patterns: (i) the first lockdown started in March 2020 and ended—following our definition—at the beginning of June 2020, i.e. in spring and summer. The second lockdown started at the beginning of November and ended in April 2021, i.e. in autumn and winter. Seasonal effects related to lifestyle, social activities, well-being, and the state of mental health might, therefore, be an influencing factor on how older people were affected by lockdown measures. (ii) The degree of uncertainty and subjective fears regarding the new illness was possibly higher in the first lockdown compared to the second. (iii) In contrast, there were much higher infection numbers and deaths from COVID-19 during the second lockdown compared to the first [27]. However, disentangling the effects of lockdown stringency and other factors such as seasonality, infection rates, or deaths from COVID-19 is not possible.

Our study confirms previous studies which showed that people living alone, women, people with NCDs, people with mental diseases and people with lower income had higher feelings of loneliness in general than their counterparts during the COVID-19 pandemic [7–12, 15, 16, 28]. The evidence of these findings is only called into question by few studies where either no effect between these subgroups could be detected, or the difference was only visible after adjusting for covariates [8, 9, 11, 14, 29].

We found little difference in loneliness between people with compulsory schooling and secondary degrees and only slightly less loneliness in people with tertiary degrees compared to the other two subgroups. These results agree with most previous longitudinal studies that found no association between educational groups and loneliness during lockdowns [9, 14, 29] with few exceptions [25].

The subgroup differences found in our study seem to be stable over all phases. Subgroups showed different levels of loneliness both during lockdown phases and before/after. Susceptible groups generally show higher levels of loneliness, but we cannot conclude that they are over-proportionately affected by lockdown measures because their levels do not follow different patterns over time. Findings from other studies are contradictory in this regard; hence, this question should be further investigated [8, 12, 14].

## Strengths & Limitations

Our study is one of the first to analyse changes in loneliness in older people over a prolonged period (21 months) of the COVID-19 pandemic. Its longitudinal structure allows us to compare changes in loneliness within individuals and with up to 20 data points during various phases of the pandemic on a very fine-grained level. Our main finding—that more stringent measures are associated with higher levels of loneliness—holds not only for the general population but also for all considered subgroups.

Of course, our study has some limitations. The fact that it is an online survey could have excluded parts of the older population with less online affinity, possibly people with lower educational levels, migrational background, or worse health. And, as with all surveys, people particularly hard hit by the pandemic and/or the measures might be less likely to participate—hence, we might underestimate adverse effects.

Our cut-off value for the Oxford stringency index for categorising more versus less stringent measures, which we placed at 55 points, could not be based on any existing predefined standard. It is higher than the median (46.1) of 27 European countries between June and August 2020, which was used by Wester et al. (2022). This study group also found an association between stringency of COVID-19 measures and feelings of loneliness in the older population [11]. Our results show that similar results can be found with a higher threshold for stringency measures.

## Conclusion

Even in a country with relatively less stringent COVID-19 measures overall like Switzerland, an increase in the proportion of older people feeling lonely could be found during phases with more stringent measures compared to phases with less stringent measures. During the course of lockdown phases, older people seems to be able to adapt to the situation somewhat, as the proportion of people feeling lonely decreased after an initial peak. Therefore, lockdown phases especially at their start should be accompanied by measures to relieve loneliness in older people.

During the first and the second lockdown, the proportion of people feeling lonely was similar, even though measures in the second lockdown were not as stringent as in the first. This suggests that there is little adaptation to lockdown measures in the course of a pandemic that could ease the effect of a second (third, etc.) lockdown. This was true, even though the two lockdown situations varied in terms of number of infections and deaths, uncertainty regarding the illness, seasonality and other factors.

Our results concerning subgroup differences align with previous findings regarding particularly susceptible subgroups: people living alone, women, people with chronic conditions or mental diseases and low income show consistently higher levels of loneliness. Although the differences are not accentuated in phases with more stringent measures, they are an important target group for interventions that could relieve loneliness to avoid adverse short- and long-term consequences.

For the older population in general and susceptible subgroups in particular,interventions to relieve loneliness during lockdowns would be important. Various interventions with this aim have been suggested in the literature [30]. These include telephone outreach, regular online group meetings or courses, help with digital inclusion and assistance programs such as delivery of meals or groceries [30]. However, most of these programs lack formal evaluation. More evidence on the effectiveness of these interventions would benefit not only older persons feeling lonely during pandemics but also those feeling lonely in normal times.
